# Boron-Related Defects in N-Type 4H-SiC Schottky Barrier Diodes

**DOI:** 10.3390/ma16093347

**Published:** 2023-04-25

**Authors:** Tihomir Knezevic, Eva Jelavić, Yuichi Yamazaki, Takeshi Ohshima, Takahiro Makino, Ivana Capan

**Affiliations:** 1 Ruđer Bošković Institute, Bijenička 54, 10000 Zagreb, Croatia; tihomir.knezevic@irb.hr; 2 Faculty of Science, University of Zagreb, Bijenička 32, 10000 Zagreb, Croatia; eva.jelavic@gmail.com; 3 National Institutes for Quantum Science and Technology, 1233 Watanuki, Takasaki 370-1292, Japan; yamazaki.yuichi@qst.go.jp (Y.Y.); ohshima.takeshi@qst.go.jp (T.O.); makino.takahiro@qst.go.jp (T.M.)

**Keywords:** silicon carbide, boron, defects

## Abstract

We report on boron-related defects in the low-doped n-type (nitrogen-doped) 4H-SiC semitransparent Schottky barrier diodes (SBDs) studied by minority carrier transient spectroscopy (MCTS). An unknown concentration of boron was introduced during chemical vapor deposition (CVD) crystal growth. Boron incorporation was found to lead to the appearance of at least two boron-related deep-level defects, namely, shallow (B) and deep boron (D-center), with concentrations as high as 1 × 10^15^ cm^−3^. Even though the boron concentration exceeded the nitrogen doping concentration by almost an order of magnitude, the steady-state electrical characteristics of the n-type 4H-SiC SBDs did not deteriorate.

## 1. Introduction

The earliest experimental studies of boron-related deep-level defects in SiC using deep-level transient spectroscopy (DLTS) date back to the 1980s. The pioneering work was performed by Anikin et al. [1] on boron-doped 6H-SiC p+n diodes. They not only reported the peak labeled as the D-center but also indicated that the D-center has two overlapping peaks with activation energies for hole emission of E_v_ + 0.63 eV and E_v_ + 0.73 eV. This result was recently confirmed experimentally using Laplace minority carrier transient spectroscopy (Laplace-MCTS) [2]. Capan et al. [2] have shown that the D-center consists of two components (i.e., emission lines) and labeled these as D_1_ and D_2_, respectively. The estimated activation energies for hole emission are E_v_ + 0.49 eV and E_v_ + 0.57 eV. They are assigned to the deep boron (isolated boron sitting at the C site) (-*h* and -*k* sites, respectively). The intensity ratio D1:D2 has been estimated at 1:1, which indicates that the B_C_(*h*) and B_C_(*k*) sites are equally occupied.

Suttrop et al. [3] have made significant progress in understanding boron-related defects, as it became evident that boron introduces two electrically active deep-level defects into SiC material. In their study, they used 6H-SiC grown by a liquid phase epitaxy (LPE) process. Boron was introduced either by ion implantation or during the LPE process from a B-doped silicon melt, and this time two electrically active deep-level defects were detected. The shallow boron at E_v_ + 0.30 eV and, already known, the deep boron (D-center) at E_v_ + 0.58 eV.

Together with aluminum, boron is the most common p-type dopant in SiC. However, unintentional incorporation of boron can occur during crystal growth. This incorporation has been previously reported and explained by the presence of boron in the graphite susceptor used for CVD growth [4,5,6]. The concentration of boron that has been unintentionally introduced during CVD growth can reach more than 10^13^ cm^−3^. Consequently, boron-related deep levels are introduced into the bandgap [2,6]. This problem was studied by Storasta et al. [7]. They applied MCTS to check the influence of unintentional boron incorporation during growth in low-doped n-type 4H-SiC. The SiC material was grown by hot-wall chemical vapor deposition (CVD). Their results also confirmed previous findings that boron introduces two deep levels, shallow (B) and deep boron (D-center), with activation energies of E_v_ + 0.27 and E_v_ + 0.67 eV, respectively. Moreover, they concluded that residual boron strongly affects the properties of SiC material grown by the CVD method. Boron-related defects act as hole traps, have large capture-cross sections, and significantly reduce the minority carrier’s lifetime.

In this work, we have performed electrical characterization employing temperature-dependent current-voltage (I–V), capacitance-voltage (C–V), DLTS, and MCTS measurements to verify the effect of unintentionally incorporated boron on the steady-state electrical performance of n-type 4H-SiC semitransparent Schottky barrier diodes.

## 2. Materials and Methods

**Figure 1:**
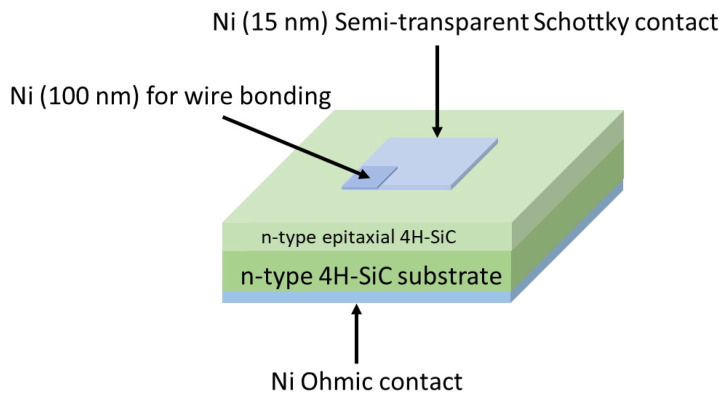
The schematic diagram of the semitransparent SBD used for MCTS measurements.

In this work, Schottky barrier diodes (SBDs) were fabricated on nitrogen-doped 4H-SiC epitaxial layers with a thickness of approximately 25 μm. The n-type epi-layer was grown on an 8° off-cut silicon face of a 350 μm thick 4H-SiC (0001) wafer without a buffer layer by CVD [8]. Semitransparent SBDs for MCTS measurements were formed by the evaporation of a thin film of nickel (15 nm) through a metal mask with openings of 2 mm × 2 mm. For wire bonding, a thick nickel film (100 nm) was stacked on one corner of the Ni thin film. Figure 1 shows the schematic diagram of the fabricated semitransparent SBDs. More information about SBDs can be found elsewhere [2]. The same semitransparent diodes were used for all experimental techniques. No high-temperature annealing was performed before electrical characterization.

The fabricated semitransparent SBDs were evaluated by I–V and C–V measurements using a Keithley 4200 SCS (Keithley Instruments, Cleveland, OH, USA). Electrically active defects were characterized by DLTS (electron traps) and MCTS (hole traps). DLTS and MCTS measurements were performed using the following setup: a Boonton 7200 capacitance meter and a NI PCI-6251 DAQ. For DLTS measurements, the following voltage settings were applied: reverse bias voltage, V_R_ = −10 V; pulse bias, V_P_ = −0.1 V. The pulse duration, t_P_, was 10 ms. For MCTS measurements, a 365 nm LED (ThorLabs M365D2 LED) with a Thorlabs LDC205C LED driver was applied. For the depth-profiling measurements, the reverse bias voltage was varied from −10 V to −1 V. More details on MCTS measurements are given elsewhere [9,10,11,12].

## 3. Results and Discussion

**Figure 2:**
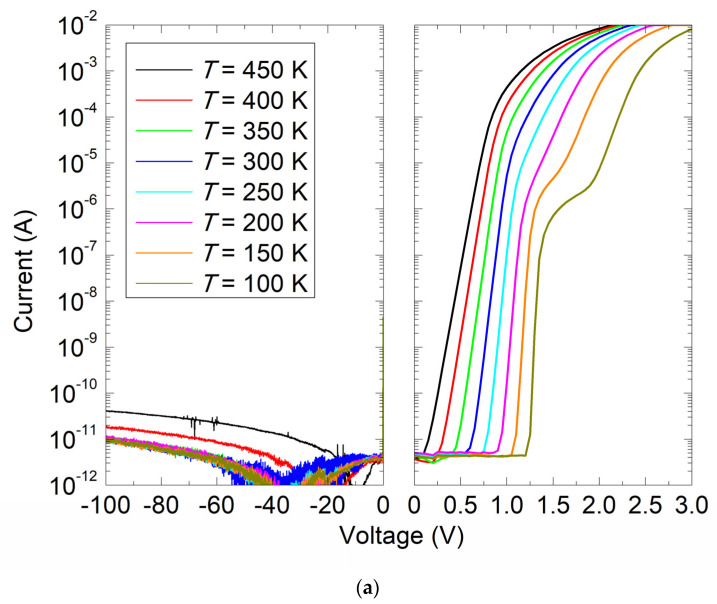

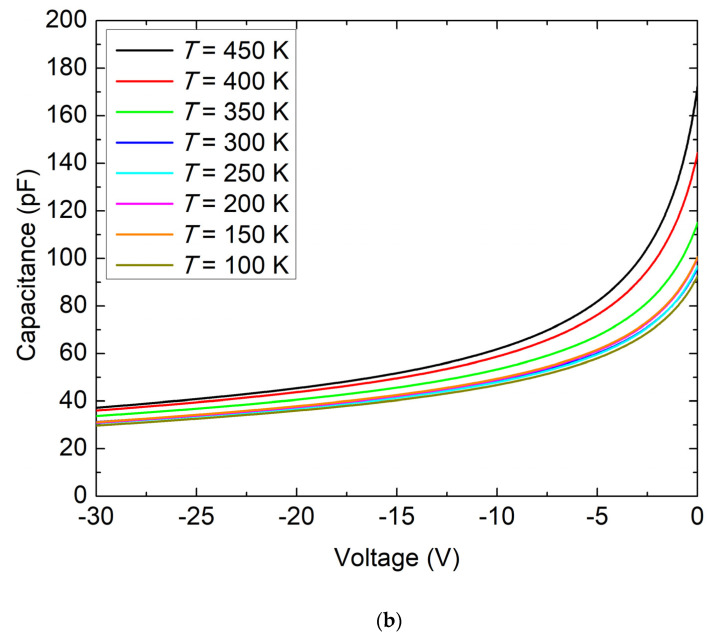
(**a**) I–V and (**b**) C–V characteristics for as-grown n-type 4H-SiC SBD measured at temperatures from 100 K to 450 K.

The steady-state electrical properties of the fabricated n-type 4H-SiC SBDs were checked by I–V and C–V measurements. Figure 2a,b show, respectively, the I–V and C–V characteristics at temperatures from 100 K to 450 K. To prevent any potential damage to the device due to high current flow, the maximum diode current was limited to 10^−2^ A. The sensitivity of the measurement setup and instrumentation is limited to a minimum current of ~1 × 10^−11^ A, and the current measured in that range will be affected by the noise floor of the equipment. The ideality factor was extracted from the slope of the linear part of the semi-logarithmic I–V curve, and at 300 K, a value of ~1.01 was obtained.

The forward I–V characteristics at temperatures below 200 K revealed two distinct regions separated by a kink that is attributed to the presence of a Schottky barrier inhomogeneity affected by surface defects, doping inhomogeneities, and local effects at the metal-semiconductor interface [13,14,15]. Barrier inhomogeneities result in the formation of multiple Schottky barrier heights, two for the studied diode, leading to deviations in the electrical performance of the diode visible through the formation of kinks. Large-area diodes, such as the one studied in this paper, are more susceptible to the formation of barrier inhomogeneities due to the higher probability of encountering surface defects and doping inhomogeneities. At higher temperatures, only the dominant Schottky barrier determined the saturation current, which remained below 50 pA even for a reverse bias voltage of 100 V and at the operating temperature of 450 K. The low leakage current of 4H-SiC SBD indicates the absence of generation-recombination mechanisms, even for a fully depleted epitaxial layer, that could otherwise degrade the electrical performance. Apart from the effect of temperature on series resistance, the C–V characteristics measured at 1 MHz and at temperatures from 100 K to 450 K show no impact of additional charge carriers on the capacitance.

**Figure 3:**
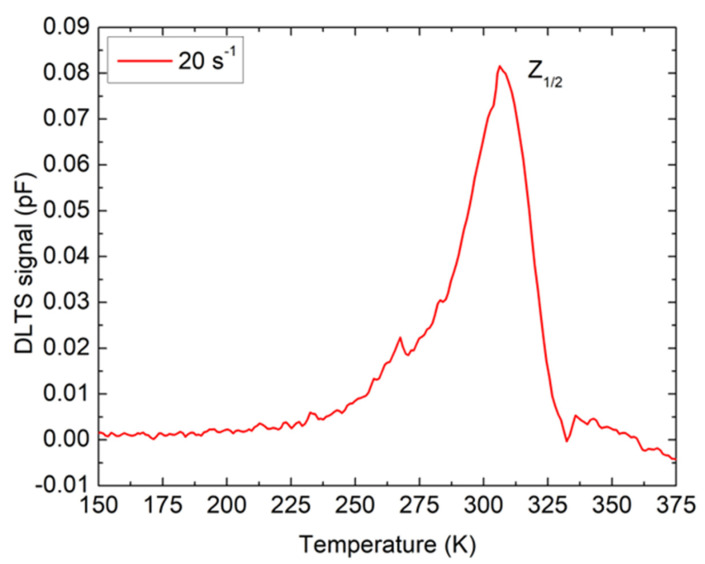
DLTS measurements for n-type 4H-SiC SBD. Measurement settings were V_R_ = −10 V, V_P_ = −0.1 V, and t_P_ = 10 ms.

Only one peak with a temperature maximum of around 310 K is observed in the DLTS spectrum (Figure 3). The peak, labeled as Z_1/2_, was already assigned to a transition between the double negative and neutral charge states of carbon vacancy V_C_ (=/0) [16,17,18]. Carbon vacancy is often labeled a “lifetime killer” due to its detrimental effect on carrier lifetime [19,20,21], and it is the most studied defect in 4H-SiC material. The activation energy for electron emission was estimated from the Arrhenius plot as Ec−0.65 eV. We also estimated the Z_1/2_ concentration to be 3 × 10^12^ cm^−3^. The obtained value is compatible with all previously published results for the low-doped n-type 4H-SiC SBDs [22,23].

Since the unknown boron concentration was unintentionally incorporated into the SiC material during CVD growth, there was a need for an experimental technique that will enable us to study the lower part of the bandgap, i.e., the minority carrier traps. It is worth noting that minority carrier traps are studied to a smaller extent compared to majority carrier traps in SiC, even though they could play an even more notable role as “lifetime killers” than the widely known Vc [7,24].

**Figure 4:**
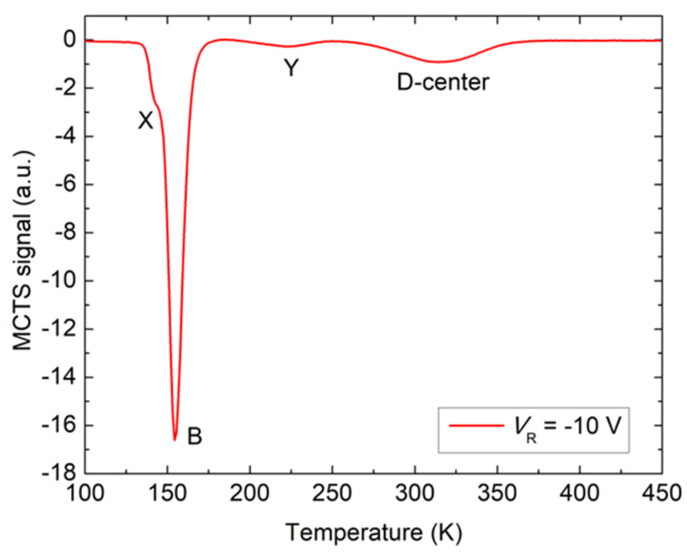
MCTS measurement for n-type 4H-SiC SBD. The voltage settings were V_R_ = −10 V.

In this work, we carried out MCTS measurements to investigate the presence of boron in n-type 4H-SiC material. The MCTS spectrum for as-grown 4H-SiC SBD is given in Figure 4. The boron concentration was high enough to introduce two known boron-related deep-level defects, B and D-center. Two smaller peaks, labeled X and Y, are observed in the MCTS spectrum with the B-center and D-center. The activation energy for hole emission for these defects could not be accurately calculated, but comparison shows that the X resembles the defect recently reported by Fur et al. [25]. They estimated the activation energy for X as E_V_ + 0.195 eV. For Y, the activation energy as well as the origin are still unclear. Further studies are needed to find out if X and Y are boron-related defects or not.

From the Arrhenius plots, the activation energies for hole emissions for B and D-center are estimated as E_V_ + 0.21 and E_V_ + 0.60 eV, respectively. They are well-known defects and have already been assigned to shallow boron (substitutional boron at the silicon site, B_Si_) and deep boron (substitutional boron at the carbon site, B_C_) [2,7,24,26,27].

The intensity of the B peak is striking (Figure 4), and it is evident that the shallow boron concentration (B) is not only significantly higher compared to deep boron (D-center), but to Z_1/2_ (Figure 3) as well. Since shallow boron occupies Si-sites, and deep boron occupies C-sites in the 4H-SiC materials, our results clearly indicate that SiC grew under C-rich growth conditions since many empty Si-sites were available for boron [28].

**Figure 5:**
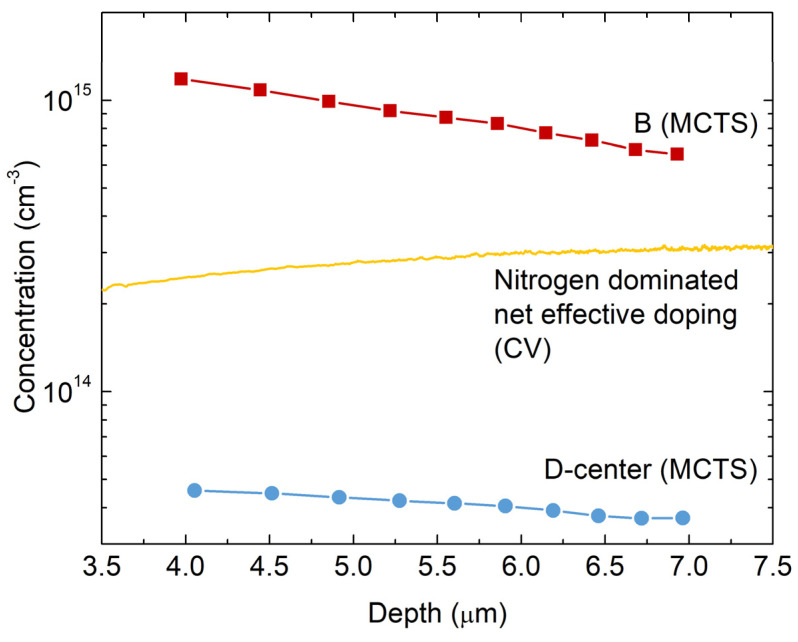
Depth profiles for B-center and D-center in n-type 4H-SiC SBD measured by MCTS. The net effective doping concentration (nitrogen-dominated) profile was estimated from C–V measurements at room temperature (RT).

We conducted depth profiling measurements using the C–V and MCTS to acquire additional information on boron incorporation and distribution. Figure 5 shows concentration profiles as a function of depth for B-center and D-center, as well as the free carrier concentration profile. The net effective doping concentration profile, *N_TOT_* = *N_D_* − *N_A_*, is calculated using:

(1) $$N_{TOT}\left(W\right)=\frac{2}{2\varepsilon_{SiC}\varepsilon_{0}A^{2}d\left(1/C^{2}\right)/dV}$$

where *ε_SiC_* is the dielectric constant for 4H-SiC of 9.7, *ε*_0_ is the vacuum permittivity, and *A* is the area of the device.

The concentration of both the B-center and D-center is increasing towards the surface. Moreover, the concentration of shallow boron, B, is more than an order of magnitude higher than the concentration of deep boron, D-center. This is consistent with the previously mentioned C-rich CVD growth conditions, where boron easily finds empty Si-sites near the surface.

Yang et al. [6] studied the correlation between the introduced boron concentration and the concentration of the D-center using MCTS and secondary-ion mass spectrometry (SIMS) measurements. They found that the ratio between the D-center and the boron concentration is 0.02. If we take this ratio and the estimated concentration of the D-center from Figure 5 as 4.5 × 10^13^ cm^−3^, we obtain 2.25 × 10^15^ cm^−3^ as the boron concentration in the SiC material. This is almost an order of magnitude higher than the nitrogen doping concentration (Figure 5) and an order of magnitude higher than the concentration of unintentionally introduced boron during CVD growth, as previously assumed [2,4,6]. It is noteworthy that such a high boron concentration did not affect the steady-state electrical properties of the n-type 4H-SiC SBDs, as we show in Figure 2.

Another interesting feature is boron diffusion in SiC, which is extremely important from a technological aspect. Most of the available studies agree that the fast diffusion of shallow baron (*B_Si_*) is explained by two mechanisms, the interstitial-mediated and the kick-out mechanisms [29]:

$$\begin{array}{c} B_{Si}↔B_{i}+V_{Si}\\ B_{Si}+Si_{i}↔B_{i}, \end{array}$$

where *B_Si_*, *B_i_*, *V_Si_*, and *Si* denote substitutional boron at the Si-site, interstitial boron, silicon vacancy, and silicon interstitial, respectively.

Regarding deep boron (B_C_), Bockstedte et al. [30] described the preferential formation of deep boron in diffusion tails and suggested that the appearance of deep boron is more favorable compared to shallow boron under nonequilibrium conditions.

From Figure 5, we can also see a slight difference in the slope of the measured depth profiles. While the concentration of D-center is more uniformly distributed over the measured volume (4 × 10^13^ cm^−3^ < [D] < 5 × 10^13^ cm^−3^), the concentration of B changes more rapidly (1.2 × 10^15^ cm^−3^ < [B] < 6 × 10^14^ cm^−3^). The volume examined is rather limited, and the changes in concentrations are not too significant; therefore, we cannot draw definite conclusions. For accurate and complete diffusion analysis, high-temperature (1700–2100 °C) annealing and SIMS measurements are prerequisites. However, the observed dependencies in the depth profiles could be tentatively explained by different diffusion mechanisms for shallow boron (*B_Si_*) and deep boron (B_C_), as described above.

## 4. Conclusions

MCTS measurements provided direct evidence that unintentional boron incorporation during CVD growth has resulted in the appearance of at least two boron-related deep-level defects, shallow boron (B) and deep boron (D-center). These defects have already been assigned to substitutional boron occupying the Si-site (*B_Si_*, shallow boron) and the C-site (B_C_, deep boron). The estimated boron concentration (~2 × 10^15^ cm^−3^) has exceeded the nitrogen doping concentration (~3 × 10^14^ cm^−3^); however, the steady-state electrical performance of the n-type 4H-SiC SBDs has been preserved.

## Data Availability

Data is contained within the article.
